# Endothelial Gene Expression and Molecular Changes in Response to Radiosurgery in *In Vitro* and *In Vivo* Models of Cerebral Arteriovenous Malformations

**DOI:** 10.1155/2013/408253

**Published:** 2013-10-02

**Authors:** Jian Tu, Zhiqiang Hu, Zhongbin Chen

**Affiliations:** ^1^Australian School of Advanced Medicine, Macquarie University, 2 Technology Place, North Ryde, Sydney, NSW 2109, Australia; ^2^Department of Neurosurgery, The 9th Medical Clinical College of Beijing University, Beijing 100850, China; ^3^Department of Electromagnetic and Laser Biology, Beijing Institute of Radiation Medicine, Beijing 100038, China

## Abstract

Radiosurgery for cerebral arteriovenous malformations (AVMs) is limited to 2-year latency. There is no early marker to monitor whether the lesion is responsive to radiosurgery. In this study, we examined endothelial gene expression and molecular changes in response to radiosurgery. Gene expression of E- and P-selectin, ICAM-1, PECAM-1, VCAM-1, tissue factor, and vWF in human cerebral microvascular endothelial cells was quantified by RT-qPCR at different radiation doses and time points. Soluble E- and P-selectin, ICAM-1, VCAM-1, and tissue factor in an animal model of AVMs were quantified by ELISA at different time after radiosurgery. We found that gene expression of E- and P-selectin, ICAM-1, PECAM-1, and VCAM-1 was upregulated by radiation in a dose-dependent manner (*P* < .05). Gene expression of E- and P-selectin and ICAM-1 was more sensitive to irradiation than that of PECAM-1 and VCAM-1. Radiosurgery induced gene expression of P-selectin, ICAM-1, PECAM-1, and VCAM-1 was linearly correlated with time (*P* < .05). Radiosurgery induced elevation of soluble E- and P-selectin, ICAM-1, VCAM-1, and tissue factor in a rat model of AVMs (*P* < .05). Thus, a combination of these molecules measured at different time points may serve as an early predictor of responsiveness of AVMs to radiosurgery.

## 1. Introduction

Cerebral arteriovenous malformations (AVMs) consist of tangles of immature vessels, which are one of the major causes of hemorrhagic stroke in children and young adults. Current treatment options include surgery, endovascular embolization, and radiosurgery. Surgery is only performed when the operative risk is lower than the morbidity and mortality associated with the natural history of the lesion. Embolization is mainly applied to reduce the size of the AVM nidus prior to definitive surgical or radiosurgical management. Radiosurgery offers a valuable addition to the treatment armamentarium for those patients who are either unable to undergo surgical resection or unable to be accessed with endovascular catheters for endovascular embolization of AVMs. Radiosurgery induces vasoocclusion, a process that involves a combination of cellular proliferation and intravascular thrombosis. It takes 2.4 years on average to occlude all vessels within the lesion [1]. Partial vasoocclusion does not reduce the risk of AVM rupture [2]. 

Vasoocclusion can occur as early as 4 months or as late as 5 years after radiosurgery or may never occur by the end of the follow-up period [3–6]. To date, angiography is the gold-standard for confirming obliteration and is performed 2-3 years after radiotherapy [5]. Early partial response to radiosurgery is difficult to be angiographically assessed in a reliable manner. An early marker that reflects AVM vessel responses to irradiation is needed to predict the effectiveness of radiosurgery. 

Following radiosurgery, AVM endothelial cells undergo apoptosis release IL-1*β* which acts as an autocrine signal to enhance endothelial cell apoptosis and also as a paracrine signal to induce expression of endothelial adhesion molecules and proinflammatory cytokines on surrounding endothelial cells [7]. Expression of adhesion molecules including E-selectin, P-selectin, ICAM-1, PECAM-1, and VCAM-1 is upregulated following irradiation [8]. E-selectin is exclusively expressed on the surface of activated endothelial cells [8] and is a critical mediator of leukocyte rolling—the first step in the cascade of events that lead to firm adhesion and transmigration into the inflamed tissues [9]. The mechanism of induction of E-selectin expression is likely to be via activation of NF*κ*B by reactive oxygen intermediates generated by irradiation [10]. P-selectin is stored in the Weibel-Palade bodies of endothelial cells. Like E-selectin, P-selectin is a central mediator of leukocyte rolling during inflammation and is also able to mediate the adherence of platelets to neutrophils and monocytes [11], allowing this molecule to participate in the cross-talk between the inflammatory and thrombogenic processes. In addition to its role during inflammation, P-selectin also participates in coagulation by binding with tissue factor to accelerate the formation and deposition of fibrin during thrombogenesis [11]. ICAM-1 is constitutively expressed on the cell surface of endothelial cells and leukocytes and functionally activates leukocyte-endothelial adhesion and migration [12]. The mechanism of induction of ICAM-1 appears to be similar to that of E-selectin with activation of NF*κ*B upregulating transcription of the ICAM-1 gene [13]. The expression of PECAM-1 is restricted to cells of the vascular system. The molecule is highly expressed at the lateral junctions of endothelial cells and on the surface of platelets and leukocytes [14]. PECAM-1 is required in the initial formation of endothelial cell-cell contacts [15] and involved in thrombosis [15]. VCAM-1 is constitutively expressed on endothelial cells [16] and mediates tethering and rolling of lymphocytes [17] and monocytes [18].

Similar to the process of endothelial apoptosis leading to altered inflammatory properties, the coagulative properties are also upregulated via increased expression of thrombotic molecules including von Willebrand factor (vWF) [19] and tissue factor [20]. vWF is synthesized in endothelial cells and stored in Weibel-Palade bodies. Radiation increases constitutive synthesis of vWF and exocytosis of the Weibel-Palade bodies. Endothelial secretion is the principal source of vWF in the plasma. vWF plays a critical role in platelet adhesion and coagulation as well as being important in leukocyte recruitment during inflammation [21]. Circulating vWF acts as a carrier protein for factor VIII and prolongs the half-life of factor VIII in the circulation by inhibiting the inactivation of factor VIII by factor Xa [22]. vWF, that is, released during thrombus formation, prevents rapid clearance of factor VIII from the plasma, thereby promoting thrombin generation [23]. Tissue factor is the primary initiator of the extrinsic coagulation cascade, and it is not expressed on endothelial cells but present on subendothelial cells and in the vascular adventitia. Under normal conditions, tissue factor is anatomically sequestered from the blood. In response to endothelial damage or cytokine stimulation, tissue factor is activated [24] and forms a complex with factor VIIa. The tissue factor-factor VIIa complex catalyses the activation of factor IX and factor X, leading to the conversion of prothrombin to thrombin and thence to the production of fibrin from fibrinogen [22, 24]. Both blood-borne tissue factor and tissue factor present in the vessel wall contribute to the initiation and propagation of the processes. Tissue factor-rich microparticles circulate within the blood and serve as a major source of blood-borne tissue factor [11]. 

Based on our previous work that endothelial molecular changes observed in human cerebral AVMs, an animal model of AVMs, and a murine endothelial cell model in response to radiosurgery [25–31], we hypothesize that expression of endothelial molecules could be upregulated at transcriptional level and detectable in blood after radiosurgery. The objectives of this study were to test our hypothesis in a human cerebral microvascular endothelial cell model as no biopsy human AVM specimens that well responded to radiosurgery treatment are possibly obtained, and an AVM animal model to establish a time course of endothelial molecule changes and identify early predictors of the ultimate response to radiosurgery.

## 2. Materials and Methods

### 2.1. Chemicals, Primers, Reagents, and Cell Culture

All chemicals, M199 and RPMI1640 phenol red free media, and cell culture supplements were purchased from Sigma-Aldrich (St. Louis, MO, USA) unless otherwise specified. All primers were synthesized by Sigma-Aldrich. Fetal calf serum (FCS) and Superscript kit were purchased from Invitrogen (Carlsbad, CA, USA). Endothelium growth factor and MTT assay kit were purchased from Roche Applied Science (Indianapolis, IN, USA). RNeasy total RNA extraction mini kit was purchased from QIAGEN (Hilden, Germany). SYBR green reaction master mix was purchased from Applied Biosystems (Darmstadt, Germany). Rat E- and P-selectins, ICAM-1, PECAM-1, VCAM-1, von Willebrand factor, and tissue factor ELISA kits were purchased from Uscn Life Science and Technology (Wuhan, China). Human cerebral microvascular endothelial cells were purchased from Applied Biological Materials (Richmond, BC, Canada) and maintained in M199 medium supplemented with endothelium growth factor, and 10% FCS in a humidified atmosphere of 37°C and 5% CO_2_. Anti-rat caspase-3 antibody was purchased from Abcam (Cambridge, UK). Alexa Fluor 488 conjugated anti-rabbit secondary antibody was purchased from Molecular Probes (Eugene, OR, USA).

### 2.2. Cell Viability Assay following Irradiation

To evaluate radiation effect on cell viability and select irradiation dose for the following experiments, human cerebral microvascular endothelialcells were examined by MTT assay following irradiation as previously reported [27, 32]. Briefly, human cerebral microvascular endothelial cells were seeded in 96 well plates coated with haemaccel (Boehringer Pharma, Amsterdam, Netherlands) at a density of 8 × 10^3^ for 48 hours. When cells were 60% confluent, the cell medium was replaced by RPMI1640 phenol red free medium supplemented with 10% FCS and endothelium growth factor. The cell cultures were then exposed to radiation delivered by an X-Knife linear accelerator (Radionics, Burlington, MA, USA) with a dose of 5, 15, or 25 Gray. Sham controls were treated identically but were not exposed to radiation.

MTT was used to determine an index of cellular viability and mitochondrial metabolic activity. MTT was prepared at 5 mg/mL in RPMI1640 phenol red free medium. The assay activity was determined at 6, 12, 24, 72, and 120 hours after irradiation and performed in triplicate and repeated twice. At each of these time points, 10 *μ*L of MTT was added to each well and incubated for 4 hours at 37°C. One hundred *μ*L of formanzan lysis was then added to each well and incubated for another 4 hours at 37°C. The absorbance of the solubilized formanzan was measured at 570 nm using a microplate reader (iMark, Bio-Rad, Hercules, CA, USA). The viability of cells in irradiation sham controls was considered as 100%.

### 2.3. Quantification of Gene Expression following Irradiation

When human cerebral microvascular endothelial cells were 60% confluent, the cells were irradiated by the X-Knife linear accelerator (Radionics) with a dose of 5, 15, or 25 Gray. Sham controls were treated identically but did not receive radiation. Total cellular RNA was isolated from the cells using RNeasy total RNA extraction mini kit (QIAGEN, Hilden, Germany) according to the manufacture's protocol. The isolated RNA was reverse-transcribed to cDNA using the Superscript kit (Invitrogen) following the manufacturer's instructions. Standard real-time PCR analysis of cDNA was performed by using SYBR green reaction master mix (Applied Biosystems) and the primers listed in Table 1 at 60°C to 95°C for 45 cycles in the Sequence Detection System (ABI Prism 7000; Applied Biosystems) following the manufacturer's instructions. The amount of PCR products was normalized using a housekeeping gene (*β*-actin or GAPDH) to calculate the relative expression ratios. Each experiment was performed in triplicate and repeated at least twice. Values were compared with those of the control-RNA obtained from sham irradiated cells. The fold-changes of the expression were calculated using threshold cycle (Ct) values. 

### 2.4. AVM Animal Radiosurgery

Animal experimentation was approved and performed in accordance with the guidelines of the corresponding institutional Experimental Animal Care and Ethics Committee and the Code of Practice for the Care and Use of Animals for Scientific Purposes [33]. AVM rat model was surgically created by an anastomosis of the left common carotid artery to the left external jugular vein as detailed in our previous reports [25, 26, 28, 34].

Sprague-Dawley male rats (7 weeks old, 230 ± 9 g) were housed in a parasite-free environment. Standard rat cages were used to house three rats per cage, and bedding was changed twice a week. Food and water were provided *ad libitum*. Rats were allowed to acclimatize to new surroundings before the experiment began. Surgical procedures were not performed in the presence of other rats. General anaesthesia was induced using a mixture of 4% isoflurane and oxygen (2 L/min) via a nose cone. The depth of anaesthesia was assessed using the respiratory rate and by checking the hind limb withdrawal to pain. No procedure was commenced until there was a consistent absence of response to pain. A heating blanket was used for the duration of the procedure.

The procedure was performed in a sterile field using aseptic technique. The left common carotid artery (CCA) was exposed, and blood flow was measured through the CCA using a 1 mm Doppler ultrasonic probe (Transonic Systems, Ithaca, NY, USA). The left external jugular vein (EJV) was then exposed and ligated with 10/0 nylon suture at its junction with the subclavian vein. An aneurysm clip was placed across the rostral EJV. Microclips were also applied proximally and distally on the CCA, and a small arteriotomy made on the lateral aspect. An end-to-side anastomosis of the EJV to the CCA was performed using a continuous 10/0 nylon suture. The clips were sequentially removed from the EJV, distal CCA, and proximal CCA. Blood flow was measured through the proximal CCA and the vein using 1 and 2 mm Doppler ultrasonic probes (Transonic Systems Inc., Ithaca, NY). The wound was closed, and isoflurane was turned off, allowing the animal to breathe oxygen until the time of awakening. Once awake, the animal was placed in an individual cage, and housed singly for one week postoperatively. Observations were carried out daily for the first week and then weekly thereafter. Observations included weight, assessment of motor function, behavior, and wound health.

Six weeks after surgery, radiosurgery was performed under intramuscular ketamine and midazolam anaesthesia. The depth of anaesthesia was assessed using the respiratory rate and by checking the hind limb withdrawal to pain. Once sedated, the animal was placed on the stage attached to the head ring of the X-Knife linear accelerator (Radionics). Correct positioning of the animal relative to the planned treatment location was confirmed by ensuring that the fistula was placed at the intersection of the three targeting laser beams that designate the centre of the radiation delivery arcs. The same treatment plan was used for all animals, giving a maximal dose of 25 Gy to the “nidus” in the subcranial region. The sham radiation controls were treated identically but did not receive radiation.

### 2.5. Immunohistochemistry

At the completion of the experiment, animals were anaesthetised and perfused with 4% paraformaldehyde. Vascular tissue from the neck region bilaterally was harvested, including proximal carotid artery, carotid-jugular anastomosis, distal carotid artery, arterialized feeding vein, AVM nidus, and draining vein. Specimens were embedded in tissue freezing medium (ProSciTech, QLD, Australia) with liquid nitrogen for immunohistochemistry. Sections were stained immunohistochemically as previously described [25, 26, 28–30]. Briefly, sections were washed in PBS at 37°C to remove tissue freezing medium. Nonspecific binding was blocked by 10% horse serum. Anti-rat caspase-3 antibody was applied and incubated at 4°C overnight. Slides were washed and incubated with Alexa Fluor 488 conjugated anti-rabbit secondary antibody for 2 hours in dark, and coverslipped and examined using a confocal microscope (Leica SP5, Germany) and imaging data analyzed using Leica LAS AF software. Fluorescence intensity units (FIU) were corrected using primary antibody controls. The FIU has been quantified as mean gray value.

### 2.6. ELISA

The assays were performed according to manufactures' instruction. Serum or plasma samples were diluted 10 to 20-fold and incubated on the ELISA plate precoated with specific rabbit anti-rat antibody. After washing, the peroxidase-labelled anti-rabbit antibodies were added. Finally, the activity of bound enzyme in all kits was determined with tetramethylbenzidine/hydrogen peroxide substrate. Absorbance was measured with an automated microplate reader (iMark, Bio-Rad Laboratories) at 450 nm. Subtraction readings at 570 nm reference wavelength from the readings at 450 nm correct for optical imperfections in the plate. The duplicate readings were averaged for each standard, and sample, subtract the average zero standard optical density. A standard curve was generated from each assay using the Microplate Manager 6 software (Bio-Rad Laboratories). The sample concentrations read from the standard curve was multiplied by the dilution factor. The intraassay and inter-assay precisions were carefully controlled within 5% and 7%, respectively.

### 2.7. Data Analysis

Data are expressed as means ± SE (number of experiments). The distribution of the studied parameters between irradiated and sham-irradiated animals was evaluated using Pearson's chi-square (*χ* ^2^) test for ELISA results. Statistical difference between groups was determined using the unpaired two-tailed *t*-test. When there were more than two groups, differences were analyzed using analysis of variance if the variances were equal and the Mann-Whitney nonparametric test if variances were unequal [35]. Linear regressions were calculated using a statistical computer package, Number Cruncher Statistical Systems [35]. A value of *P* < .05 was considered statistically significant.

## 3. Results

### 3.1. Cell Viability

**Figure 1:**
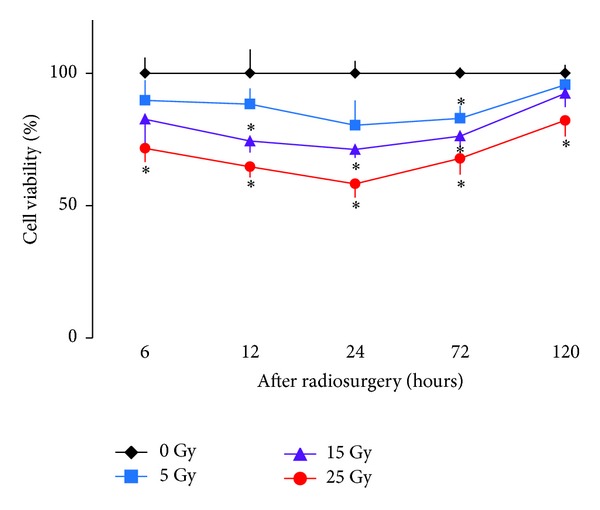
Effect of radiosurgery on cell viability. Mitochondrial metabolic activity of human cerebral microvascular endothelial cells was determined by MTT assay as an indicator of cell viability following radiosurgery at 0, 5, 15, or 25 Gray at 6, 12, 24, 72, and 120 hours. *P < .05 versus 0 Gray by paired comparison at the same time point.

Radiation effect on the cell viability of human cerebral microvascular endothelial cells was assessed as shown in Figure 1. The cell viability of sham irradiation controls was considered as 100%. At 6-hour post-irradiation, a range of radiation dosages from 0 to 15 Gray had no significant effect on the cell viability. The cell viability of human cerebral microvascular endothelial cells declined by 28 ± 2% at 25 Gray 6 hours after irradiation (*P* < .05). At 12 or 24 hours after irradiation, there was no significant effect on the cell viability at a dose of 5 Gray. The cell viability reduced by 25 ± 2% and 35 ± 2% at 15 and 25 Gray (*P* < .05) 12 hours after irradiation, respectively. Further decline in the cell viability was observed at 24 hours after irradiation, being 28 ± 2% and 41 ± 3% at 15 and 25 Gray (*P* < .05), respectively. At 72 hours after irradiation, there was a significant reduction in the cell viability at 5, 15, or 25 Gray. The cell viability recovered by 13–15% in groups that received 5, 15, or 25 Gray at 120-hour post-irradiation. The level of cell viability in the group that received 25 Gray remained significantly lower than that of sham irradiation controls (*P* < .05).

### 3.2. Apoptosis of AVM Vessels *In Vivo*

**Figure 2:**
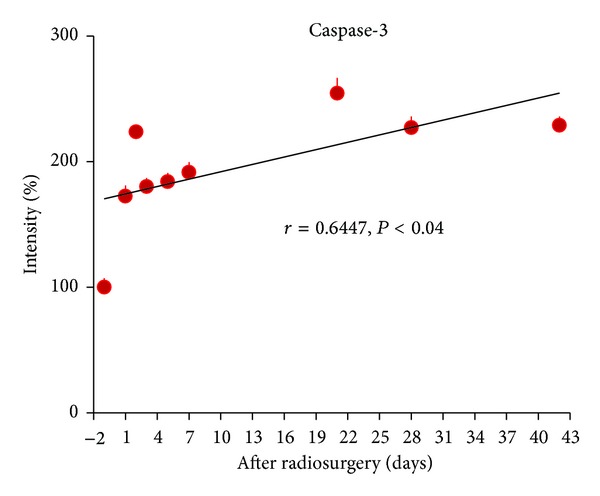
Apoptosis of AVM vessels induced by radiosurgery. Caspase-3 was examined immunohistochemically in AVM vessels before and after radiosurgery at 25 Gray. The intensity of immunofluorescence of AVM vessels was quantified using a confocal microscope. The intensity of immunofluorescence of AVM vessels observed before irradiation was arbitrarily assigned as 100%. Data were expressed as means ± SE of 4 rats at each time point. There was a positive correlation between the intensity of immunofluorescence of AVM vessels and time (*r* = 0.6447, *P* < .04), suggesting that apoptosis of AVM vessels was increasing with time over a period of 42 days after radiosurgery.

Caspase-3 is a key enzyme in apoptotic signaling pathway and is selected as a marker for apoptosis. The levels of caspase-3 expression in an animal model of AVMs over a period of 42 days after radiosurgery at 25 Gray were shown in Figure 2. There was a significant upregulation of caspase-3 expression in AVM vessels after irradiation. The levels of overexpression of caspase-3 were 72% at 1 day after irradiation and peaked at 154% at 21 days after radiation. There was a positive correlation between caspase-3 levels and time over a period of 42 days after radiosurgery (*r* = 0.6447, *P* < .04).

### 3.3. Radiosurgery Induced Gene Expression *In Vitro*

**Figure 3:**
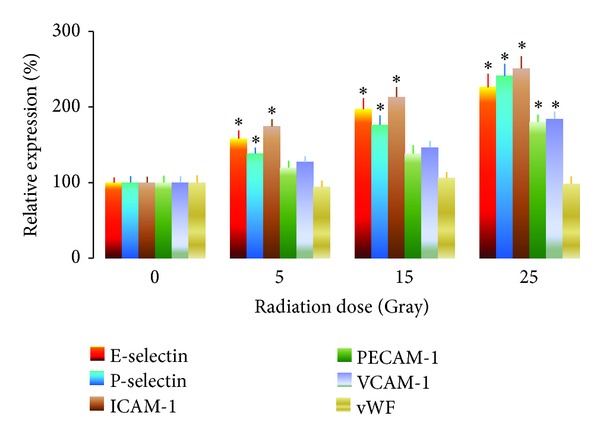
Radiosurgery induced dose-responsive endothelial gene expression in human cerebral microvascular endothelial cells. Endothelial gene expression at 0 Gray irradiation was arbitrarily assigned as 100%. Relative gene expression of E- and P-selectin and ICAM-1 was upregulated at 5, 15, or, 25 Gray. Relative gene expression of PECAM-1 and VCAM-1 was upregulated at 25 Gray. *P < .05 versus the same gene at 0 Gray.

Radiosurgery induced endothelial gene expression was dose-responsive as shown in Figure 3. At 5 Gray, the relative gene expression of E- and P-selectin and ICAM-1 was significantly increased by 58 ± 4%, 38 ± 3%, and 74 ± 3% (*P* < .05), respectively. At 15 Gray, the relative gene expression of E- and P-selectin and ICAM-1 was significantly increased by 97 ± 7%, 76 ± 6%, and 113 ± 7% (*P* < .01), respectively. At 25 Gray, the relative gene expression of E- and P-selectin, ICAM-1, PECAM-1, and VCAM-1 was significantly increased by 126 ± 9%, 141 ± 8%, 151 ± 8%, 80 ± 5%, and 84 ± 5% (*P* < .05), respectively. There was no change in vWF gene expression before and after irradiation. Twenty-five Gray was selected for experiments to determine the time course of radiation induced endothelial gene expression.

**Figure 4:**
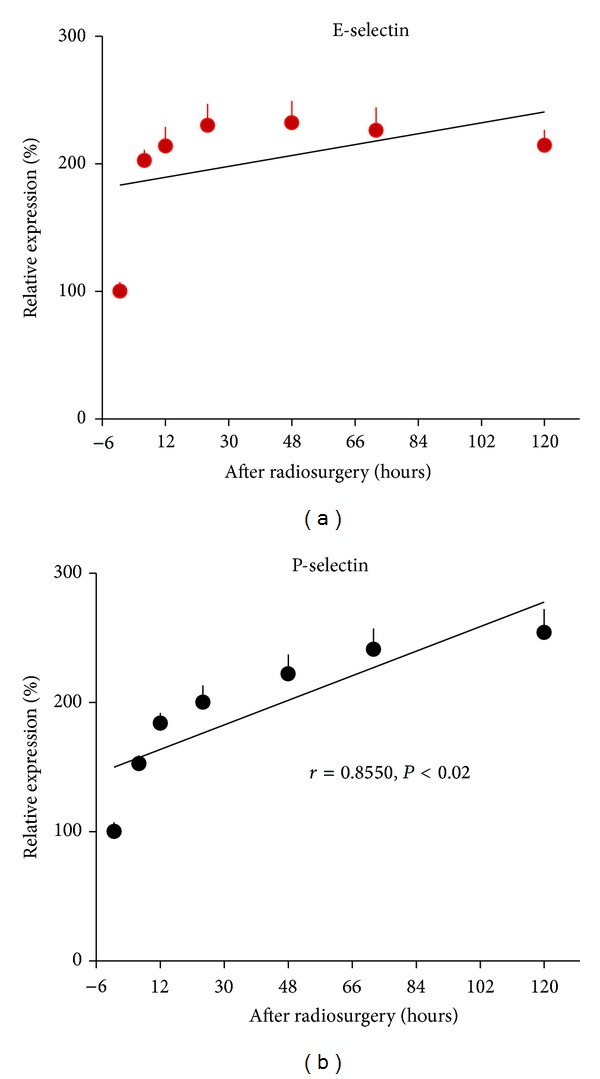
Radiosurgery induced E- and P-selectin gene expression in human cerebral microvascular endothelial cells. (a) E-selectin gene expression was doubled at 6, 12, 24, 48, 72, and 120 hours after radiosurgery (*P* < .01). (b) There was a positive correlation between P-selectin gene expression and time over 120 hours after radiosurgery (*r* = 0.855, *P* < .02).

**Figure 5:**
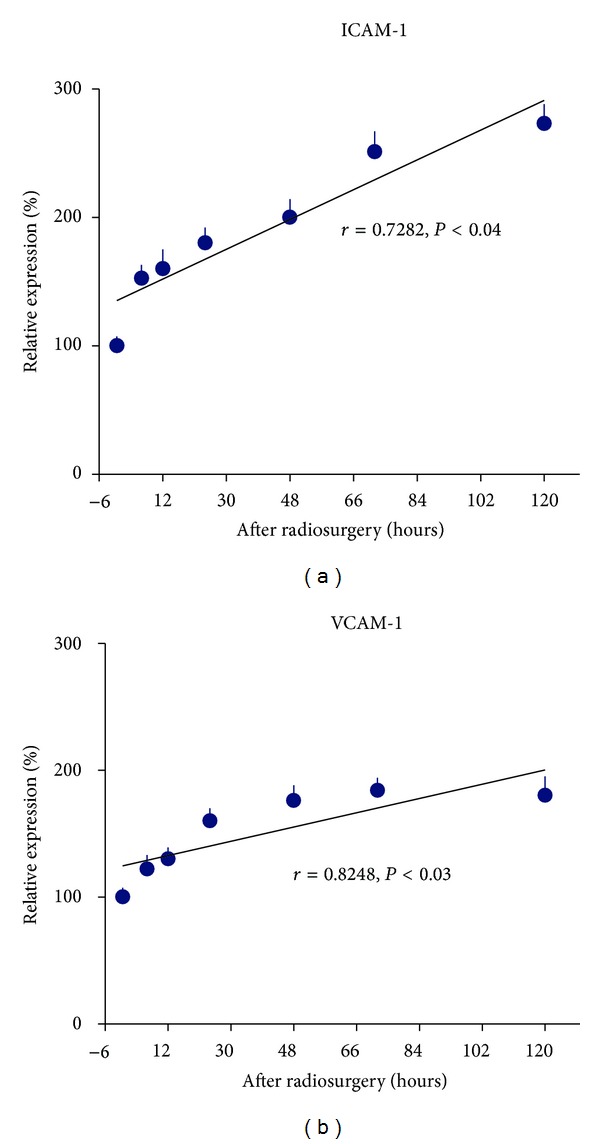
Radiosurgery induced ICAM-1 and VCAM-1 gene expression in human cerebral microvascular endothelial cells. (a) ICAM-1 gene expression was increased with time over 120 hours after radiosurgery (*r* = 0.7282, *P* < .04). (b) There was a positive correlation between VCAM-1 gene expression and time over 120 hours after radiosurgery (*r* = 0.8248, *P* < .03).

**Figure 6:**
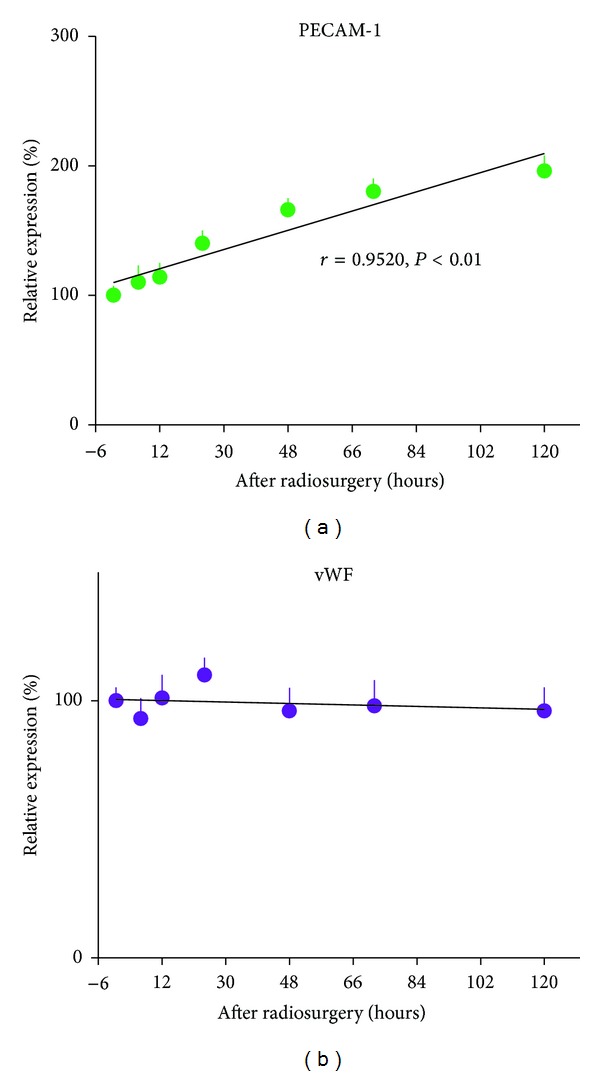
Radiosurgery induced PECAM-1 and vWF gene expression in human cerebral microvascular endothelial cells. (a) PECAM-1 gene expression was increased with time over 120 hours after radiosurgery (*r* = 0.952, *P* < .01). (b) There was no radiation responsive vWF gene expression in human cerebral microvascular endothelial cells.

Radiosurgery induced E- and P-selectin gene expression was shown in Figure 4. Radiation induced E-selectin gene expression was increased more than 1-fold over 120 hours, and peaked at 48 hours after irradiation. Radiation induced P-selectin gene expression was increased by 1.5-fold at 120 hours after radiosurgery, and the levels were positively correlated with time over 120 hours (*r* = 0.855, *P* < .02). Radiosurgery induced gene expression of ICAM-1 and VCAM-1 was shown in Figure 5. ICAM-1 gene expression was increased by 1.7-fold at 120 hours after irradiation, and a positive correlation was observed between gene expression levels and time (*r* = 0.7282, *P* < .04). VCAM-1 gene expression peaked at 72 hours after radiosurgery, and was positively increased with time (*r* = 0.8248, *P* < .03). Radiosurgery induced gene expression of PECAM-1 and vWF was shown in Figure 6. PECAM-1 gene expression was doubled at 120 hours after irradiation, and a positive correlation between PECAM-1 gene expression and time was observed (*r* = 0.952, *P* < .01). No radiation responsive vWF gene expression was observed in human cerebral microvascular endothelial cells.

### 3.4. Radiosurgery Induced Soluble Molecule Changes *In Vivo*

**Figure 7:**
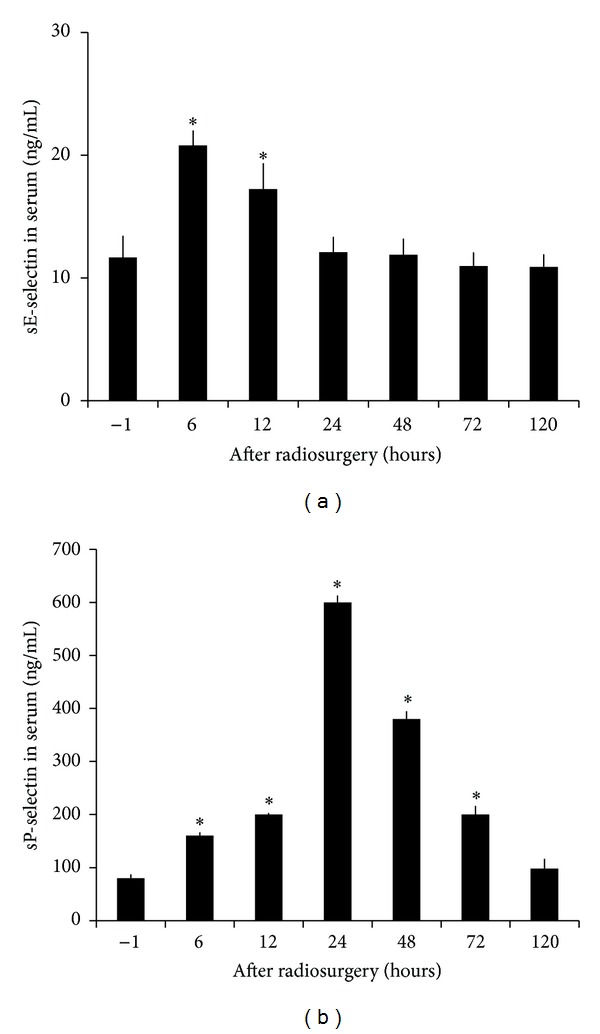
Radiosurgery induced soluble E- and P-selectin changes in AVM rat serum. (a) Serum concentrations of soluble E-selectin were significantly increased at 6 and 12 hours after radiosurgery (*P* < .05). (b) Serum levels of soluble P-selectin were significantly increased at 6, 12, 24, 48, and 72 hours after radiosurgery (*P* < .05).

**Figure 8:**
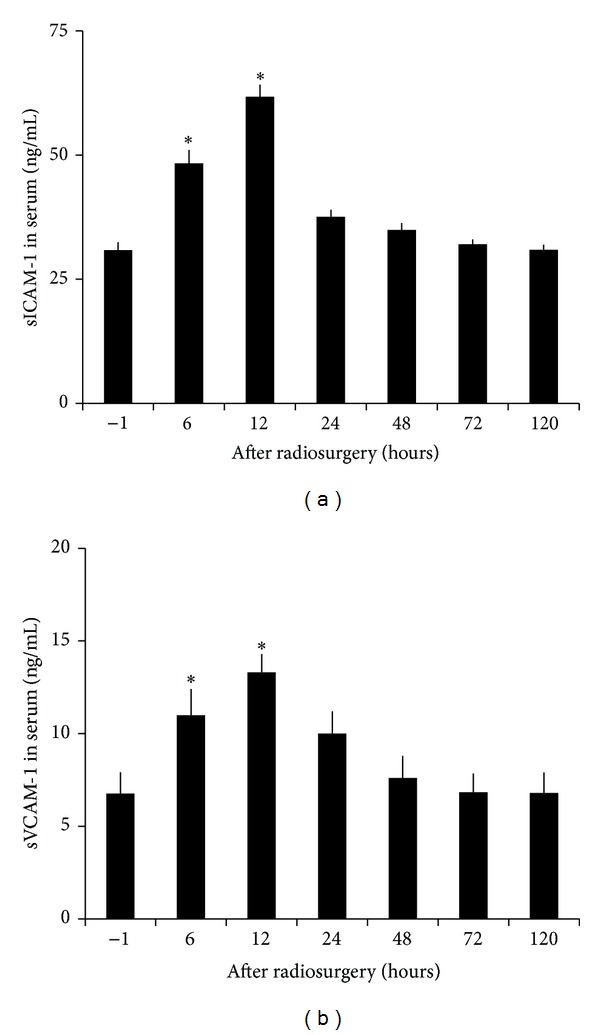
Radiosurgery induced soluble ICAM-1 and VCAM-1 changes in AVM rat serum. (a), (b) Serum concentrations of soluble ICAM-1 and VCAM-1 were significantly increased at 6 and 12 hours after radiosurgery (*P* < .05), respectively.

**Figure 9:**
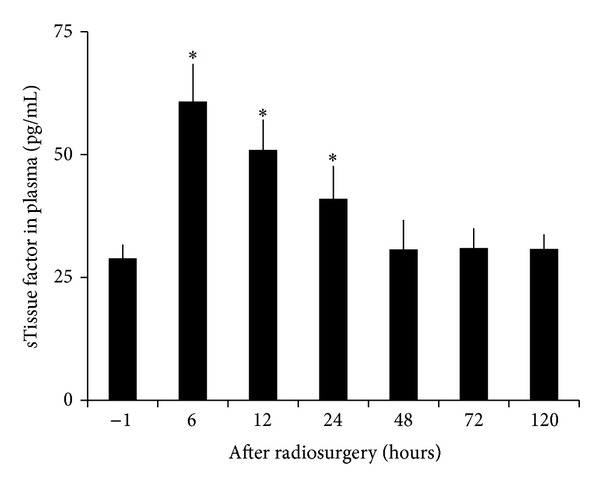
Radiosurgery induced soluble tissue factor changes in AVM rat plasma. Plasma concentrations of soluble tissue factor were doubled at 6 hours after radiosurgery and maintained at high levels for another 18 hours (*P* < .05) before returning to the baseline.

Radiosurgery inducedserum soluble E- and P-selectin changes in AVM model were shown in Figure 7. Soluble E- and P-selectin were inducible by irradiation. Serum levels of soluble E-selectin doubled at 6 hours after irradiation and returned to baseline at 24 hours. Soluble P-selectin was increased by 5-fold and 4-fold 24 and 48 hours after irradiation, respectively. Such high serum levels of soluble P-selectin returned to baseline at 120 hours after radiation. Radiosurgery induced serum soluble ICAM-1 and VCAM-1 changes in AVM model were shown in Figure 8. Soluble ICAM-1 was increased by 57 ± 2% in serum 6 hour after radiation (*P* < .05) and doubled within another 6 hours, before returning to the baseline at 120 hours. Soluble VCAM-1 was increased by 63 ± 1% in serum 6 hours after irradiation (*P* < .05), doubled within another 6 hours, and maintained at 48 ± 1% increase at 24 hours before returning to the baseline at 120 hours. Radiosurgery induced plasma soluble tissue factor changes in AVM model were shown in Figure 9. Soluble tissue factor was increased by 110 ± 6%, 76 ± 4%, and 42 ± 4% in plasma at 6, 12, and 24 hour after irradiation (*P* < .05), respectively, before returning to the baseline at 120-hour.

## 4. Discussion

Based on our previous immunohistochemistry and transmission electron microscopy studies that radiosurgery induces endothelial molecular changes in human cerebral AVM specimens, an animal model of AVMs, and a mouse cerebral endothelial cell model [25–31], we hypothesize that endothelial molecules could be upregulated at transcriptional level, detectable in serum or plasma after radiosurgery, and possibly serve as early predictors for whether AVM vessels are responsive to radiosurgery treatment. The current study has achieved the following findings.

- Human cerebral microvascular endothelial cells are much more sensitive to irradiation than those microvessels dilated to form an AVM nidus following arteriovenous fistula formation in rats.
- Expression of E- and P-selectin, ICAM-1, PECAM-1, and VCAM-1 is upregulated by radiation at transcriptional level and in a dose-dependent manner (Figure 3). Gene expression of E- and P-selectin and ICAM-1 are more sensitive to irradiation than that of PECAM-1, and VCAM-1. Radiosurgery induced gene expression of P-selectin, ICAM-1, PECAM-1, and VCAM-1 is linearly correlated to time (Figures 4–6).
- Radiosurgery induces significant elevation of serum soluble E- and P-selectin, ICAM-1, VCAM-1, and plasma soluble tissue factor in a rat model of AVMs. Timing of the measurement of soluble endothelial molecules after radiation is critical (Figures 7–9).

### 4.1. Radiosurgery Induces Inflammatory Molecule Expression

Although a single dose highly focused irradiation reduces the viability of human cerebral microvascular endothelial cells (Figure 1) and induces apoptosis of AVM vessels in a rat model (Figure 2), vascular endothelial cells remain metabolically active and able to upregulate their inflammatory molecule expression in a dose-dependent (Figure 3) and time-dependent manner (Figures 4–6(a)). Radiosurgery induced serum concentration changes of E- and P-selectin, ICAM-1, PECAM-1, and VCAM-1 are time-dependent (Figures 7 and 8). The molecular substrates for radiosurgery induced inflammatory response appear to include E- and P-selectin, ICAM-1, and VCAM-1. E-selectin, acting together with P-selectin, mediates the slow rolling of leukocytes on the endothelium leading to firm adhesion [36, 37]. VCAM-1 plays an important role in lymphocyte homing and migration [17]. E-selectin, ICAM-1, and VCAM-1 are stimulated by other factors that are present in the AVM vessels [38]. These stimuli may act synergistically with radiation to further up-regulate E-selectin, ICAM-1, and VCAM-1, thereby increasing the specificity of expression. Upregulation of E- and P-selectin, ICAM-1, and VCAM-1 is the earliest demonstrable effects of radiation. They collectively mediate the rolling, arrest and transmigration of leukocytes on the endothelium of AVM vessels [25, 30]. Radiosurgery induced inflammatory response plays an important part in a multitude of processes, including endothelial cell death, cellular neoproliferation, and thrombosis in AVM vessels [39]. 

### 4.2. Radiosurgery Induces Thrombotic Molecule Expression

Radiosurgery induces thrombus formation in human AVM vessels [40, 41]. Radiation may influence the expression of a number of molecules that play important roles in coagulation and thrombosis, including tissue factor, vWF, and P-selectin [19, 20, 42, 43]. As expected, tissue factor gene was not detected in human cerebral microvascular endothelial cells as it does not express on endothelial cells and is normally present on subendothelial cells and in the vascular adventitia of vessels [44]. Following radiosurgery of AVM vessels in a rat model, vessels lose their endothelial lining [25, 26, 28], resulting in exposure of subendothelial cells to the luminal surface, which release tissue factor directly to blood circulation; thus, plasma levels of soluble tissue factor become detectable and were upregulated in a time-dependent manner (Figure 9). The presence of exposed tissue factor to the luminal contents is associated with occlusive thrombus formation [25, 26, 28]. Exposure of subendothelial tissue factor would be important in the induction of thrombosis in AVM vessels; therefore, plasma levels of soluble tissue factor could be an early predictor of the ultimate response to radiosurgery. 

The levels of radiosurgery induced endothelial gene expression and molecular changes observed in the current study are systematically more accurate than those in irradiated human AVM specimens reported in our previous immunohistochemistry study [30]. The possible explanations are that human AVM specimens were surgically collected from patients at least 2 years after radiosurgery, and they were not responding to irradiation. Soluble forms of vWF and PECAM-1 were not measured as they appear to lose the potential to be an early responsive predictor to radiosurgery. Further experiments in AVM patients undergoing radiosurgery may help to delineate the clinical value of these endothelial molecules. Nevertheless, this current study provides useful data that may lead to the development of biomarker for monitoring early responsiveness of radiosurgery. 

## 5. Conclusions 

This study provides strong evidence that radiosurgery induced gene expression of P-selectin, ICAM-1, PECAM-1, and VCAM-1 in human cerebral microvascular endothelial cells was linearly correlated with time and elevation of soluble E- and P-selectin, ICAM-1, VCAM-1, and tissue factor in AVM vessels of a rat model. Thus, a combination of P-selectin, ICAM-1, VCAM-1, and tissue factor measured at different time points may serve as an early predictor of responsiveness of AVMs to radiosurgery.

## Figures and Tables

**Table 1:** Sequences of human gene-specific primers used for quantitative RT-PCR analysis.

Primers	Forward 5′→3′	Reverse 5′→3′
E-selectin	CATGGCTCAGCTCAACTT	GCAGCTCATGTTCATCTT
P-selectin	CAGTGGCTTCTACAACAGGC	TGGGTCATATGCAGCGTTA
ICAM-1	GCAGACAGTGACCATCTACAGCTT	CTTCTGAGACCTCTGGCTTCGT
PECAM-1	CCAGTGTCCCCAGAAGCAAA	TGATAACCACTGCAATAAGTCCTTTC
VCAM-1	GGCAGGCTGTAAAAGAATTGCA	GTCATGGTCACAGAGCCACCTT
Tissue factor	CCCAGGCAGTCAGATCATCTTCT	GCACCCAATTTCCTTCCATTT
vWF	TAAGAGGGCAACACAAACG	ATCTTCACCTGCCCACTCC
GAPDH	AGCTGAACGGGAAGCTCACTGG	GGAGTGGGTGTCGCTGTGAAGTC
*β*-actin	CTGGCACCCAGCACAATG	CCGATCCACACGGAGTACTTG

## Abbreviations

AVM: Arteriovenous malformations
CCA: Common carotid artery
Ct: Threshold cycle
EJV: External jugular vein
ELISA: Enzyme-linked immunosorbent assay
FCS: Fetal calf serum
FIU: Fluorescence intensity units
GAPDH: Glyceraldehyde-3-phosphate dehydrogenase
ICAM-1: Intercellular adhesion molecule-1
IL-1*β*: Interleukin-1 beta
MTT: 3-(4,5-dimethylthiazol-2-yl)-2,5-diphenyltetrazolium bromide
NF*κ*B: Nuclear factor kappa-light-chain-enhancer of activated B cells
PECAM-1: Platelet/endothelial cell adhesion molecule-1
RT-qPCR: Real-time quantitative polymerase chain reactions
VCAM-1: Vascular cell adhesion molecule-1
vWF: von Willebrand factor.
